# Design and Development of a Bioink for Fabricating Crosslinked Hydrogel Microneedles via 3D Printing for Transdermal Delivery of Estradiol Nanoparticles

**DOI:** 10.3390/pharmaceutics18070772

**Published:** 2026-06-24

**Authors:** Southamany Sisavengsouk, Teeratas Kansom, Boonnada Pamornpathomkul, Porawan Aumklad, Tanasait Ngawhirunpat, Praneet Opanasopit, Phuvamin Suriyaamporn

**Affiliations:** 1 Pharmaceutical Development of Green Innovations Group (PDGIG), Division of Industrial Pharmacy, Faculty of Pharmacy, Silpakorn University, Nakhon Pathom 73000, Thailand; sisavengsouk_s@su.ac.th (S.S.); kansom_t@su.ac.th (T.K.); pamornpathomkul_b@su.ac.th (B.P.); ngawhirunpat_t@su.ac.th (T.N.); opanasopit_p@su.ac.th (P.O.); 2 Research and Innovation Center for Advanced Therapy Medicinal Products, Faculty of Pharmacy, Silpakorn University, Nakhon Pathom 73000, Thailand; 3 OLIC (Thailand) Limited, Bang Pa-in, Ayutthaya 13160, Thailand; aumklad_p@su.ac.th

**Keywords:** photopolymerizable bioinks, 3D-printed microneedles, estradiol valerate, nanoparticles, transdermal delivery

## Abstract

**Background**: Conventional transdermal drug delivery systems are often limited by poor skin permeability and low drug loading efficiency, necessitating the development of advanced delivery platforms. **Objectives**: This study aimed to develop and optimize photopolymerizable bioinks (PBs) for liquid crystal display (LCD)-based 3D printing of crosslinked hydrogel microneedles (cHMNs) to enhance transdermal delivery of estradiol valerate (E2V). **Methods**: A Box–Behnken design (BBD) was used to optimize the effects of Gantrez™ S-97, Jurymer™, and polyvinyl alcohol (PVA) on viscosity, exposure time, hardness, and elasticity, with strong predictive performance (R^2^ = 0.9702–0.9907). Results: Estradiol valerate-loaded nanoparticles (E2V-NPs) were prepared via ionotropic gelation, exhibiting a particle size of 698.33 (0.78) nm, PDI of 0.50 (0.06), zeta potential of −39.09 (7.32) mV, and high encapsulation efficiency (86.87 (0.78)%). The optimized PBs enabled fabrication of uniform cHMNs (~800 µm height) with adequate mechanical strength (hardness 20.45 (1.23) N; elasticity 2.97 (0.49) MPa) and effective insertion capability. The E2V-NPs-loaded cHMNs exhibited sustained drug release over 12 days (~56.92 (4.27)%). Skin permeation studies showed a significantly enhanced flux (10.81 (4.55) µg/cm^2^/h) and cumulative permeation (12.94 (2.06) µg/cm^2^) compared to topical E2V-NPs and suspension, along with increased skin accumulation (38.55 (0.10) µg). Cytotoxicity studies confirmed that E2V and E2V-NPs were biocompatible (>80% viability), while PBs showed concentration-dependent cytotoxicity. **Conclusions**: Overall, this integrated platform combining design of experiment, nanoparticles, microneedles, and LCD 3D printing offered a promising strategy for enhancing transdermal drug delivery efficiency and reproducibility.

## 1. Introduction

Transdermal drug delivery systems (TDDSs) have revolutionized how medications are administered, offering a non-invasive alternative to oral and injectable routes. By delivering drugs directly through the skin, these systems can provide a controlled release of medication into the bloodstream, enhancing therapeutic efficacy and patient compliance [1,2]. TDDSs bypass the gastrointestinal tract and first-pass metabolism in the liver, which can degrade certain medications and reduce their effectiveness. This method not only improves bioavailability but also minimizes side effects associated with fluctuating drug levels. However, despite their numerous advantages, transdermal drug delivery systems also face significant limitations. One of the primary challenges is the barrier function of the skin. The outermost layer of the skin, the stratum corneum, is highly effective at preventing the penetration of foreign substances, including drugs [3,4]. This barrier restricts the types of drugs that can be effectively delivered through the skin, typically limiting them to those with low molecular weight and high lipophilicity. Additionally, variations in skin permeability between individuals and different skin sites can affect the consistency and predictability of drug absorption. To overcome these barriers, various enhancement techniques, such as chemical enhancers, iontophoresis, and microneedles, are being explored [5]. Advances in materials science and drug formulation have further expanded the potential of TDDSs, enabling the delivery of a wider range of therapeutics. As research continues, transdermal drug delivery holds promise for even more innovative treatments, offering a patient-friendly and efficient approach to medication administration [6,7].

Microneedles (MNs) for TDDSs represent a groundbreaking advancement in drug administration, combining the advantages of transdermal systems with enhanced drug delivery capabilities. MNs are tiny, needle-like structures that are micron-scale technology (approximately 100 to 1000 µm), designed to penetrate the outermost layer of the skin painlessly, the stratum corneum. By creating microchannels in the skin, these MNs facilitate the delivery of drugs that would otherwise be too large or too hydrophilic to cross the skin barrier effectively. Moreover, their minimally invasive nature reduces the risk of infection and improves patient compliance through self-application [8,9,10]. The development of MN technology addresses several limitations associated with conventional transdermal drug delivery systems. Traditional methods are often restricted to small, lipophilic drugs due to the formidable barrier properties of the stratum corneum. However, MNs can deliver a broader range of therapeutic agents, including hydro-/lipophilic drugs, proteins, peptides, vaccines, and other macromolecules, by bypassing this barrier. This approach enhances drug bioavailability and enables a more consistent and predictable drug release profile [11,12].

MN fabrication methods are at the forefront of advancing transdermal drug delivery technology, enabling precise and efficient drug administration through the skin. These methods are designed to produce high-accuracy MNs, ensuring their effectiveness and safety for medical applications. The choice of fabrication technique is crucial and depends on the desired MN material, size, shape, and intended use. Commonly used techniques include lithography-based methods, micro-molding, laser cutting, electroforming-electrodeposition, and 3D printing [8,13,14]. Lithography-based methods, such as photolithography and soft lithography, are widely used to create detailed MN molds. Micro-molding techniques allow for the production of MNs from various materials, including polymers, metals, and ceramics, by filling molds with the desired substance and solidifying it. Laser cutting and etching provide a high degree of precision, enabling the creation of MNs with intricate designs and sharp tips. Electroforming and electrodeposition involve the deposition of metal ions onto a mold or substrate to form MNs. Additionally, innovative manufacturing or 3D printing is increasingly employed for its ability to construct MNs layer by layer, offering customization and rapid development. These fabrication methods, combined with advances in materials science, continue to expand the potential of MNs in transdermal drug delivery, making them a versatile and effective tool in modern medicine. Three-dimensional printing offers significant advantages over traditional fabrication methods, including rapid prototyping, customization, reduced costs, and the ability to create complex geometries in a single process. It supports various materials and minimizes waste, making it an efficient and scalable solution. These benefits position 3D printing as a superior method for producing MNs, overcoming many challenges of traditional techniques and advancing the potential of transdermal drug delivery systems [15,16].

The fabrication of crosslinked hydrogel microneedles (cHMNs) using 3D printing is highly appropriate due to these technologies’ high resolution and ability to work with biocompatible bioinks [17,18]. Stereolithography (SLA) [19], digital light processing (DLP) [20], and liquid crystal display (LCD) [21] are commonly employed to fabricate MNs. While all three techniques utilize photopolymerization to build objects layer by layer, their light sources and methods of projecting the pattern onto the photopolymerizable bioinks (PBs) differ. SLA uses a laser to trace each layer, DLP projects the entire layer image at once using a digital projector, and LCD uses an LCD screen to mask the UV light, simultaneously curing the layer as a whole [22,23]. Each technique has its advantages and is chosen based on specific printing requirements such as speed, resolution, and material properties. LCD is often considered the best choice for fabricating cHMNs because it is cost-effective, offers suitable printing speed, and achieves high resolution [24,25]. PBs used in these processes are typically biocompatible polymers mixed with photoinitiators such as Irgacure^®^ 2959 (I2959), lithium phenyl-2,4,6-trimethylbenzoylphosphinate (LAP), 2,3-bornanedione (Camphorquinone), diphenyl(2,4,6-trimethylbenzoyl) phosphine oxide (TPO), 2,4,6-trimethylbenzoyl)-phenylphosphine oxide (BAPO), riboflavin, and Ru(bpy)_3_Cl_2_ or photoabsorbers such as tartrazine, methylene blue, eosin Y and Sudan I. These bioink materials offer several advantages, including the ability to produce safe, biocompatible, and biodegradable cHMNs [25,26]. Furthermore, bioinks can be compatible with drugs, allowing for the direct printing of drug-loaded cHMNs. This capability ensures that the cHMNs can effectively deliver therapeutic agents through the skin. As research advances, drug-loaded cHMNs produced by 3D printing are poised to become a pivotal technology in the future of transdermal drug delivery, offering innovative solutions for a wide range of medical applications [27]. Integrating 3D printing with cHMNs enhances the potential for creating personalized and effective drug delivery systems, ultimately improving patient outcomes and expanding the possibilities of non-invasive therapies [16].

Although bioinks have previously been explored for 3D printing of microneedles, the systematic optimization of nanoparticle-loaded PBs for LCD-based 3D printing of cHMNs remains limited. In particular, the successful fabrication of drug-loaded cHMNs requires a careful balance between formulation properties, printing performance, photocrosslinking behavior, mechanical strength, and drug delivery capability. Empirical formulation development may be insufficient to control these interdependent factors, especially when nanoparticle systems are incorporated into PB matrices. Therefore, the novelty of this study lies not simply in the use of PBs for 3D-printed microneedles, but in the integration of nanoparticle-based drug delivery with statistically optimized PB formulations for LCD-based 3D printing of cHMNs. In this work, estradiol valerate-loaded nanoparticles (E2V-NPs) were used as a model poorly soluble drug delivery system, while design of experiments (DoE), using a Box–Behnken design (BBD), was applied to systematically optimize the PB composition. This strategy allowed the relationships between formulation variables and critical performance attributes, including viscosity, exposure time, hardness, and elasticity, to be quantitatively evaluated. By combining pharmaceutical formulation science, digital manufacturing, and statistical optimization, this study provides a rational framework for developing E2V-NPs-loaded cHMNs with suitable printability, mechanical integrity, and enhanced transdermal delivery performance. The optimized platform may therefore contribute to the future development of more reproducible, customizable, and data-driven microneedle-based delivery systems for poorly soluble therapeutic agents.

## 2. Materials and Methods

### 2.1. Materials

Estradiol valerate (E2V) was purchased from Tokyo Chemical Industry (Tokyo, Japan). Lithium phenyl (2,4,6-trimethylbenzoyl) phosphinate (LAP), polyethylene glycol dimethacrylate (PEGDMA; MW = 550 Da), chitosan (CS; low MW = 50–190 kDa with 96% deacetylation), tripolyphosphate (TPP), and polyvinyl alcohol (PVA; M.W. = 85–124 kDa) were purchased from Sigma-Aldrich (Dorset, UK). Gantrez™ S-97 (GAN; Poly-methyl vinyl ether-alt-maleic acid; MW = 1500 Da) was purchased from Ashland (Surrey, UK). Jurymer^®^ (polyacrylic acid) was received from TOAGOSEI CO., LTD. (Chonburi, Thailand). N-naphthyl-N,O-succinyl chitosan was supported from national nanotechnology center (national science and technology development agency, Pathumthani, Thailand). The fresh neonatal porcine skins were received from the local slaughterhouse (Nakhon Pathom, Thailand). Phosphate-buffered saline (PBS) was prepared from 137 mM of NaCl, 2.68 mM of KCl, 10.14 mM of Na_2_HPO_4_ and 1.76 mM of KH_2_PO_4_, adjusted to pH 7.4. All other reagents and chemicals were of analytical grade.

### 2.2. Development and Optimization of Photopolymerizable Bioinks (PBs) for LCD 3D Printing Using Design of Experiment (DoE)

The PBs for 3D printing were based on PEGDMA-550, supplemented with photoinitiators and additional polymers. A 0.5% *w*/*w* solution of LAP was first dissolved in water. Subsequently, other polymers, including GAN, Jurymer, and PVA, were added and mixed for 24 h until completely dissolved, according to the DoE shown in Table 1. A Box–Behnken design (BBD) was employed to generate the experimental dataset, resulting in 15 PB formulations. Afterward, 60% *w*/*w* PEGDMA 550 was added and mixed until a homogeneous solution was obtained in an amber glass bottle under magnetic stirring for at least 2 h. The prepared PBs were then stored at room temperature prior to use. The input factors included the concentrations (% *w*/*w*) of GAN, Jurymer, and PVA, while the output responses were viscosity, exposure time, hardness, and elasticity. LAP, PEGDMA 550, and water were maintained as constant factors. A suitable statistical model was selected to describe the relationships between the variables. The optimal PB formulation was determined based on minimizing viscosity and exposure time while maximizing hardness and elasticity.

### 2.3. Viscosity Measurement

The viscosity of PBs was measured using a viscometer (Brookfield DV2T; Middleboro, MA, USA). An 8 mL sample was loaded into the viscometer receptacle, and the sensing rods (small sample adapter, SSA28/13R) were lowered into the receptacle until fully immersed in the bioinks. The samples were tested at appropriate rpm and % torque (80–100%) at 25 °C. The viscosity of each sample was recorded in centipoise (cP).

### 2.4. Exposure Time Test

The optimal exposure time of PBs for LCD 3D printing was determined under a UV wavelength of 405 nm and an approximate light intensity of 3–5 mW/cm^2^ using an LCD 3D printer (Phrozen Sonic Mini 8K, Hsinchu, Taiwan). A calibration STL file (disk-shaped, 0.5 cm in height and 1.5 cm in diameter) was used to evaluate a range of exposure times. The PBs were added into the resin tank and sequentially exposed using the LCD screen of the 3D printer. The printing parameters were set with a layer height of 22 µm, lift distance of 1 mm, and lift speed of 60 mm/min. The printed structures were visually assessed for completeness and structural integrity. The exposure time that achieved the best balance of cured properties without over-curing or under-curing was determined to be the setting for these specific PBs.

### 2.5. Compression and Tensile Test

Disk-shaped samples were fabricated using an LCD 3D printer at the optimized exposure time for each PB formulation obtained from the previous study. After printing, the samples were washed with 70% EtOH and post-cured under UV light to ensure complete polymerization prior to mechanical testing. A texture analyzer was used to evaluate the compression behavior of the crosslinked samples. The hardness of the disk-shaped samples was determined using a force–displacement test under axial compression. Each sample was placed at the center of the platform, and a cylindrical stainless-steel probe (P/1KSS, 1 cm diameter) was lowered at a constant speed of 1 mm/s. Upon contact between the probe and the sample surface, the displacement was set to zero. The force was then continuously applied until the displacement reached 0.5 cm, corresponding to the sample thickness. The force–displacement curves were recorded and used to determine the maximum hardness (maximum compressive force, N) and elasticity (stress, MPa) of each sample.

### 2.6. Fourier Transform Infrared (FT-IR)

FTIR (Nicolet iS5, diamond crystal, Thermo Fisher Scientific, Madison, WI, USA) was performed on the polymers and crosslinked samples to evaluate the crosslinking process. The FTIR spectra of individual components, including GAN, Jurymer, PVA, LAP, PEGDMA 550, and the 3D-printed disk-shaped sample, were recorded and analyzed. The spectra were collected over a wavenumber range of 400–4000 cm^−1^, with 16 scans per sample at a resolution of 4 cm^−1^, using an attenuated total reflectance (ATR) mode.

### 2.7. Preparation and Characterization of E2V-Loaded Nanoparticles (E2V-NPs)

Estradiol valerate-loaded nanoparticles (E2V-NPs) were prepared using the ionotropic gelation method, adapted from a previous study [28]. NSCS was used as the polymer, while TPP served as the ionic crosslinking agent. The formulation ratio of NSCS:TPP:E2V was fixed at 1:1.5:5 mg/mL. Briefly, E2V and NSCS were dissolved in DMSO at predetermined concentrations. The TPP solution was then added dropwise into the NSCS/E2V solution under continuous magnetic stirring at 800 rpm and 25 °C. The addition was performed using a syringe pump connected to a 24G needle at a constant rate of 0.25 mL/min for 20 min. After complete addition, the mixture was further stirred for 30 min to ensure complete ionic crosslinking and formation of NSCS/TPP nanoparticles. Subsequently, the particle size was reduced using probe sonication at 40% amplitude (20 kHz) for 10 min to obtain a uniform nanoparticle dispersion. The resulting E2V-NPs were further purified by dialysis to remove DMSO using a dialysis membrane (CelluSep^®^, MWCO 6000–8000 Da, Seguin, TX, USA).

For E2V-NP characterization, the particle size (PS), polydispersity index (PDI), and zeta potential (ZP) of the prepared nanoparticles were measured using a Zetasizer Nano Series (Malvern Instruments, Version DTS 4.10, Malvern, UK). All measurements were performed under appropriate dilution conditions. The amount of E2V encapsulated in the nanoparticles was determined by dissolving the nanoparticles in methanol to completely extract the drug. The E2V content was quantified using HPLC (Agilent 1200 Series LC Systems and Modules, Agilent Technologies, Santa Clara, CA, USA). The HPLC analysis was performed using a C18 column (4.6 × 250 mm, 5 µm) maintained at 40 °C. The mobile phase consisted of acetonitrile and ultrapure water (80:20, *v*/*v*) at a flow rate of 1 mL/min. Detection was carried out using a UV detector at 220 nm, and the injection volume was 20 µL. The entrapment efficiency (%EE) was calculated using the following Equation (1).

(1) $$\%\mathrm{Entrapment}\;\mathrm{efficiency}\;(\%\mathrm{EE})=\frac{\mathrm{Amount}\;\mathrm{of}\;E2V\;\mathrm{encapsulated}}{\mathrm{Amount}\;\mathrm{of}\;E2V\;\mathrm{added}}\times 100$$

### 2.8. Fabrication of 3D-Printed cHMNs Loaded with E2V-NPs

**Figure 1:**
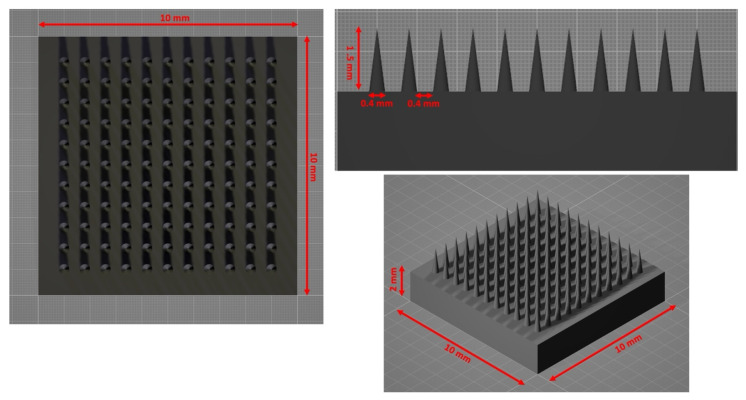
Conical 3D-printed cHMN model (11 × 11 array) with a height of 1500 µm, a base diameter of 400 µm, and an inter-needle spacing of 400 µm (patch size 10 × 10 mm^2^), created using 123D Design software.

The optimal PBs obtained from the DoE were mixed with E2V or E2V-NPs until a completely homogeneous solution was achieved. An STL file, designed to generate conical 3D-printed cHMNs (11 × 11 array) with a height of 1500 µm, a base diameter of 400 µm, and an inter-needle spacing of 400 µm (patch size 10 × 10 mm^2^), was created using Autodesk 123D Design software (version 2.2.14, Autodesk Inc., San Rafael, CA, USA), presented in Figure 1. The STL file was imported into an LCD 3D printer (Phrozen Sonic Mini 8K, Hsinchu, Taiwan) using CHITUBOX Basic software (version 2.2.0, CBD-Tech, Shenzhen, China). The PBs containing E2V or E2V-NPs were then poured into the resin tank and used to fabricate the 3D-printed cHMNs. After printing, the cHMNs were washed with 70% EtOH to remove excess PBs and subsequently cured in a UV curing chamber (450 nm) for 30 min. The 3D-printed cHMNs loaded with E2V or E2V-NPs were then dried at 40 °C for 24 h to remove residual water and enhance mechanical strength and durability, and were subsequently stored in a desiccator for further investigation.

### 2.9. Morphology Study of 3D-Printed cHMNs Loaded with E2V-NPs

The morphology of the 3D-printed cHMNs loaded with E2V-NPs was observed under a digital microscope (Dino-Lite Edge/5 MP AM7915 series; AnMo Electronics Corporation, New Taipei City, Taiwan) and a scanning electron microscope (SEM; Mira TC, TESCAN ORSAY HOLDING, a.s., Brno, Czech Republic) at appropriate magnifications. Before observation by SEM, the sample was coated with gold and used at a beam voltage of 15.0 kV. The samples were imaged and measured to assess their shape, as well as the needle height, width, and interspacing.

### 2.10. Mechanical Properties of 3D-Printed cHMNs Loaded with E2V-NPs

#### 2.10.1. Mechanical Strength

The mechanical properties of 3D-printed cHMNs and cHMNs loaded with E2V or E2V-NPs were evaluated using a texture analyzer in compression mode. Hardness was determined using a force–displacement test under axial compression. Each sample was placed at the center of the platform with the needles facing upward. A cylindrical stainless-steel probe (P/1KSS, 1 cm diameter) was lowered toward the sample at a constant speed of 1 mm/s. When the tips of the cHMNs contacted the probe, the displacement was set to zero, and the force was continuously applied until the displacement reached 1 mm, corresponding to the height of the cHMN patch. The force–displacement curves were recorded and used to determine the maximum hardness and elasticity of each cHMN.

#### 2.10.2. Penetration Study

A penetration study was conducted to evaluate the insertion capability of 3D-printed cHMNs loaded with E2V-NPs for creating microchannels in skin-like tissue. An 8-layer Parafilm^®^ M model was used to simulate human skin due to its comparable thickness. The Parafilm^®^ M layers were stacked and fixed onto a paraffin wax board. The 3D-printed cHMNs were pressed onto the Parafilm^®^ M using a texture analyzer under a compression force of 20 N for 30 s. After removal of the cHMNs, the puncture sites on each Parafilm^®^ M layer were imaged using a digital microscope. The percentage of complete penetration was calculated according to Equation (2) [29,30].

(2) $$\%\mathrm{Complete}\;\mathrm{penetration}=\frac{\mathrm{Number}\;\mathrm{of}\;\mathrm{puncture}\;\mathrm{dots}}{\mathrm{Total}\;\mathrm{needles}\;\mathrm{number}\;\mathrm{of}\;\mathrm{MNs}}\times 100$$

### 2.11. Histological Study

Histological analysis of porcine skin was performed after application of 3D-printed cHMNs loaded with E2V-NPs to evaluate penetration depth. Fresh porcine skin samples were prepared using standard histological procedures, including fixation in 10% formalin, followed by dehydration, clearing, and embedding in frozen section medium. The prepared tissues were sectioned in a cross-sectional orientation using a cryostat microtome at −35 ± 2 °C, with a thickness of 10 µm. The sections were then stained with hematoxylin and eosin (H&E) and observed under an inverted microscope. Penetration depth was determined from the histological images.

### 2.12. Drug Content Determination

The 3D-printed cHMNs loaded with E2V-NPs were dissolved in 50 mL of ethanol for 24 h. The samples were then subjected to bath sonication at 40 kHz for 30 min to facilitate drug extraction from the polymer matrix. Subsequently, the solutions were appropriately diluted with ethanol and filtered through a 0.45 µm syringe filter prior to HPLC analysis.

### 2.13. Degradation Study

Porcine skin was fixed onto a dental wax board to provide structural support. Drops (0.5 mL) of PBS (pH 7.4) containing 2.0 U/mL esterase were applied to simulate interstitial fluid. The 3D-printed cHMNs loaded with E2V-NPs were placed onto the porcine skin, and a compression force of 20 N, representing the average human pressing force, was applied using a texture analyzer for 30 s. After removal of the applied force, the cHMNs remained inserted at the center of the porcine skin and were incubated at 37 °C for predetermined time intervals (0, 2, 6, 12, and 24 h). At each time point, the cHMNs were removed, and their height was measured. The percentage of height degradation was calculated based on the reduction in cHMN height over time using the corresponding Equation (3) [31].

(3) $$\mathrm{The}\;\mathrm{percentage}\;\mathrm{of}\;\mathrm{height}\;\mathrm{degradation}\;\left(\%\right)=\frac{H_{0}-H_{1}\;}{H_{0}}\times 100$$

where *H*_0_ was the initial height of cHMNs before the degradation test, and *H*_1_ was the final height of cHMNs after degradation.

### 2.14. In Vitro Drug Release Kinetic Study

The drug release behavior of 3D-printed cHMNs loaded with E2V or E2V-NPs was investigated. Phosphate-buffered saline (PBS, pH 7.4) containing 20% ethanol was used as the release medium under sink conditions. An E2V suspension and each cHMN containing equivalent drug amounts were placed in dialysis bags and immersed in 50 mL of the release medium. The system was maintained under constant agitation at 200 rpm and a temperature of 37 ± 2 °C. At predetermined time intervals (0, 1, 2, 3, 4, 5, 6, 7, and 12 days), 1 mL of the release medium was withdrawn and replaced with fresh medium to maintain sink conditions. The collected samples were analyzed for drug content using HPLC. The release profiles were further evaluated using various kinetic models, including zero-order, first-order, Higuchi, Korsmeyer–Peppas, and Hixson–Crowell models. The most appropriate model was selected based on the highest coefficient of determination (R^2^) to describe the drug release mechanism.

### 2.15. In Vitro Skin Permeation and Drug Accumulation Study

The skin permeation study was conducted using vertical Franz diffusion cells with porcine skin. The receptor compartment was maintained at 37 ± 1 °C and filled with PBS (pH 7.4) containing 20% ethanol and was continuously stirred at 200 rpm using a magnetic stirrer. Each formulation (E2V suspension and 3D-printed cHMNs loaded with E2V or E2V-NPs), containing equivalent drug amounts, was applied to the donor compartment to evaluate drug permeation through the skin. For cHMNs, a compression force of 20 N was applied using a texture analyzer for 30 s on the porcine skin prior to mounting in the Franz diffusion cell. At predetermined time intervals (0, 0.5, 1, 2, 4, 6, 8, 10, 12, and 24 h), samples were withdrawn from the receptor compartment and replaced with fresh medium to maintain sink conditions. The collected samples were analyzed for drug content using HPLC. Following the permeation study, drug accumulation in the porcine skin was determined. The skin samples were removed and sectioned into smaller pieces, followed by extraction with ethanol. The extracts were centrifuged at 14,000× *g* for 30 min to separate tissue debris. The resulting filtered supernatant was then quantitatively analyzed using HPLC.

### 2.16. In Vitro Cytotoxicity Study

The biocompatibility of the formulations was evaluated using normal human fibroblasts (NHFs). Cell viability was assessed for E2V suspension, E2V-NPs, PBs, and PBs containing E2V-NPs with equivalent drug content using the MTT assay. NHF cells were cultured in Dulbecco’s Modified Eagle Medium (DMEM) supplemented with 10% fetal bovine serum (FBS) and incubated at 37 °C in a humidified atmosphere with 5% CO_2_. The cells were seeded into 96-well plates at a density of 10,000 cells per well and incubated until 70–80% confluency was reached. Each formulation was prepared in serum-free DMEM at various concentrations and added to the wells, followed by incubation for 24 h. After treatment, 25 µL of MTT solution (5 mg/mL) was added to each well and incubated for an additional 3 h. The medium was then removed, and the resulting formazan crystals were dissolved in 100 µL of DMSO. The optical density (OD) of each well was measured at 550 nm using a microplate reader (VICTOR Nivo™ Multimode Plate Reader, PerkinElmer, Inc., Waltham, MA, USA). Cell viability (%) was calculated according to Equation (4), with untreated cells used as the control (100% viability).

(4) $$\%\mathrm{Cell}\;\mathrm{viability}\;(\%)=\frac{\mathrm{Abs}.\;\mathrm{of}\;\mathrm{treated}\;\mathrm{group}}{\mathrm{Abs}.\;\mathrm{of}\;\mathrm{control}\;\mathrm{group}}\;\times \;100$$

### 2.17. Statistical Analysis

The DoE process was analyzed in Design Expert^®^ software version 13 (free trial version). All data were reported as the mean (standard deviation; SD). The significance of the differences was evaluated using a two-sided independent *t*-test for comparing two groups and a one-way analysis of variance (ANOVA) with a Tukey post hoc test for multiple groups. In addition, for small sample sizes (*n* < 20) where normality could not be confirmed, appropriate nonparametric tests were applied, such as Kruskal–Wallis 1-way ANOVA. The significance level was set at a *p*-value of 0.05. This statistical analysis was performed using Microsoft Excel software version 2021 with the data analysis extension.

## 3. Results and Discussion

### 3.1. Development and Optimization of Photopolymerizable Bioinks (PBs) for LCD 3D Printing Using Design of Experiment (DoE)

The BBD was successfully applied to evaluate the effects of three formulation variables, namely Gantrez S97 (X_1_), Jurymer (X_2_), and PVA (X_3_), on the critical quality attributes (CQAs) of PBs, including viscosity, exposure time, hardness, and elasticity. The results in Table 1 demonstrated a strong dependence of all responses on polymer concentration and their interactions. Notably, increasing PVA concentration (factor C) markedly elevated viscosity (up to 39,673 cP) and exposure time (up to 300 s), indicating a denser polymer network that hindered light penetration and curing efficiency [32]. Similarly, higher levels of GAN (factor A) significantly enhanced mechanical properties, particularly hardness (up to 23.43 N) and elasticity (up to 5.42 MPa), suggesting improved crosslinking density and structural integrity [33]. However, excessive polymer content led to undesirable increases in viscosity and curing time, which may negatively impact printability [34,35]. Overall, the BBD model effectively captured the nonlinear relationships and interactions among variables, enabling identification of an optimal design space that balances low viscosity and exposure time with high mechanical strength, which is critical for achieving high-resolution and mechanically robust 3D-printed microneedles.

The ANOVA and multiple regression analysis based on the BBD that reduced quadratic models were statistically significant and appropriate for describing all responses, as evidenced by *p*-values < 0.05 and non-significant lack of fit (*p* > 0.05), reported in Table 2. High coefficients of determination (R^2^ = 0.9702–0.9907) further confirmed the excellent predictive capability and reliability of the developed models. The regression equations revealed that GAN (X_1_) and PVA (X_3_) were the dominant factors influencing all responses, showing strong positive effects on viscosity (Y_1_), exposure time (Y_2_), hardness (Y_3_), and elasticity (Y_4_), with significant quadratic terms (X_1_^2^ and X_3_^2^) indicating nonlinear behavior. In contrast, Jurymer (X_2_) exhibited a comparatively smaller or negative influence, particularly on viscosity and exposure time, and contributed mainly through interaction effects such as X_2_X_3_. The positive interaction between X_1_ and X_3_ consistently enhanced multiple responses, suggesting a synergistic effect that likely increased crosslinking density and mechanical strength [36]. Notably, viscosity and exposure time increased substantially with higher polymer concentrations, which may adversely affect printability, whereas hardness and elasticity improved, reflecting enhanced structural integrity [34,35].

**Figure 2:**
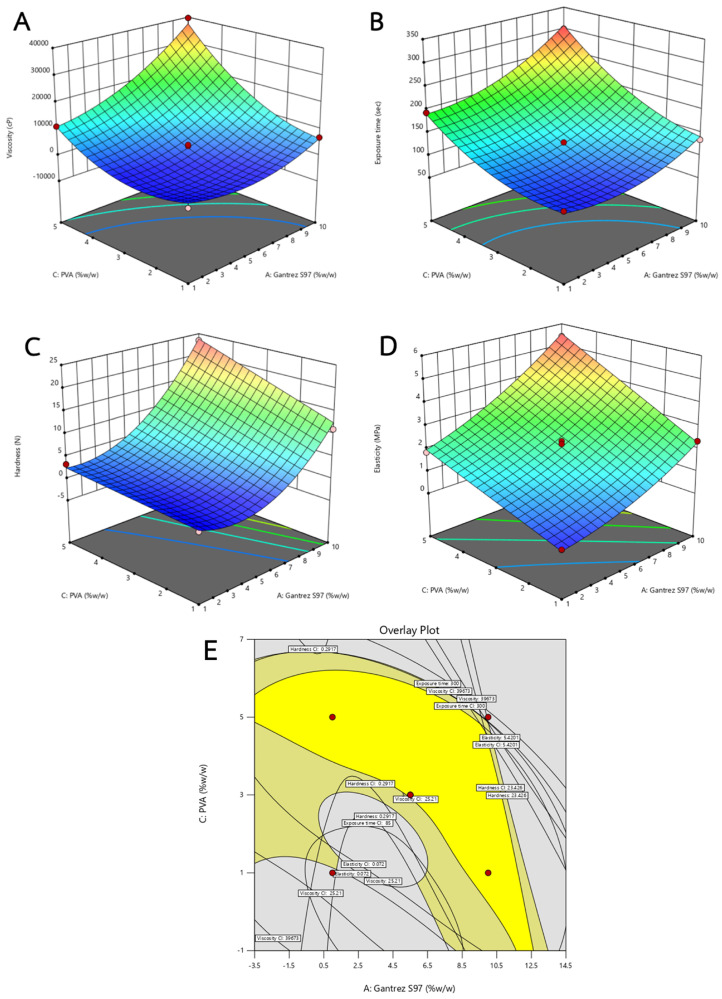
Response surface plots illustrating the effects of Gantrez S97 (X_1_) and PVA (X_3_) concentrations on the (**A**) viscosity (cP), (**B**) exposure time (sec), (**C**) hardness (N), (**D**) elasticity (MPa) and (**E**) quality control design space of photopolymerizable bioinks.

The response surface plots (Figure 2A–D) clearly illustrated the interactive effects of GAN (X_1_) and PVA (X_3_) on the critical properties of the photopolymerizable bioinks. As shown in Figure 2A, viscosity (Y_1_) increased markedly with increasing concentrations of both GAN and PVA, exhibiting a pronounced curvature that confirms the significant quadratic and interaction effects between these variables. This behavior was attributed to increased polymer chain entanglement and higher crosslinking density, which reduce flowability at higher concentrations [36,37]. Similarly, Figure 2B demonstrates that exposure time (Y_2_) followed the same trend, increasing significantly with higher levels of X_1_ and X_3_, likely due to reduced light penetration and slower curing kinetics in more viscous systems [32]. In contrast, Figure 2C,D showed that hardness (Y_3_) and elasticity (Y_4_) improved substantially with increasing GAN and PVA concentrations, indicating enhanced mechanical strength and flexibility of the crosslinked network [32,38]. The upward curvature of the surfaces suggests strong synergistic interactions, particularly between X_1_ and X_3_, which contribute to improved structural integrity of the cHMNs. However, the results also highlight a critical trade-off: while higher polymer concentrations enhance mechanical properties, they simultaneously increase viscosity and exposure time, potentially compromising printability and process efficiency [36,37]. Therefore, an optimal region can be identified at intermediate levels of GAN and PVA, where a balance between rheological properties and mechanical performance is achieved, supporting the development of high-resolution and robust 3D-printed microneedles.

The overlay plot (Figure 2E) further supports the response surface findings by identifying an optimal design space where all responses were simultaneously satisfied. Consistent with previous results, increasing GAN and PVA improved hardness and elasticity but also elevated viscosity and exposure time. The yellow region represents the feasible zone, where a balance between printability and mechanical performance was achieved. Notably, the optimal region was located at intermediate polymer concentrations, confirming the trade-off observed earlier. Outside this region, excessive polymer content leads to high viscosity and prolonged curing, while lower levels result in insufficient mechanical strength [36,37].

The optimized formulation predicted by the model demonstrated good agreement with experimental validation, confirming the reliability of the BBD and desirability approach, reported in Table 3. The optimal composition consisted of GAN (10.00% *w*/*w*), Jurymer (3.20% *w*/*w*), and PVA (2.04% *w*/*w*), with an overall desirability of 0.659, indicating a balanced compromise between competing responses. The predicted results (R_p_) for viscosity, exposure time, hardness, and elasticity were 9569.12 cP, 162.34 s, 15.48 N, and 3.162 MPa, respectively, which closely matched the experimental results (R_e_). Importantly, all observed values fell within the 95% prediction intervals, confirming the robustness and predictive accuracy of the developed models. Slight deviations between predicted and experimental values may be attributed to inherent experimental variability and process sensitivity, particularly in photopolymerization systems. Consistent with previous findings, the optimized formulation achieved a balance between acceptable viscosity and exposure time for printability, while maintaining sufficient mechanical strength [36,37]. These results validate the applicability of the developed model for guiding formulation design and ensuring reproducible performance of 3D-printed microneedles.

### 3.2. Fourier Transform Infrared (FT-IR)

**Figure 3:**
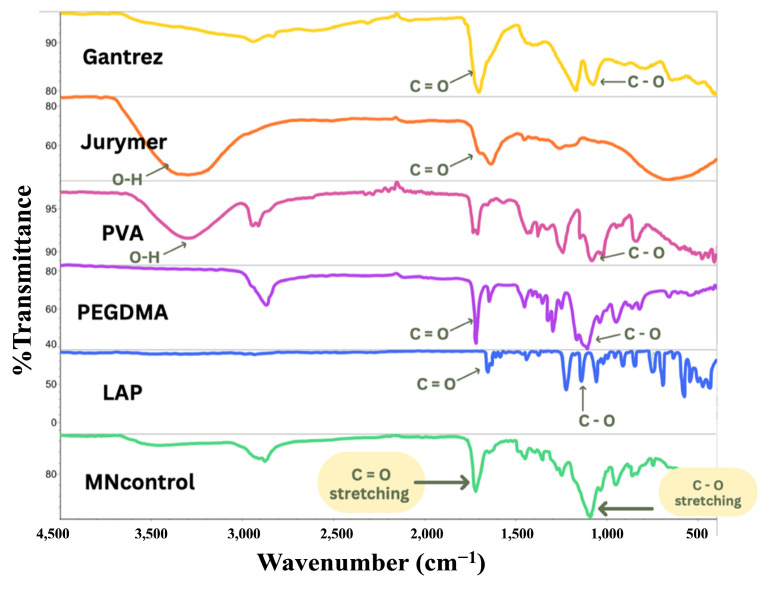
FTIR analysis of individual components and 3D-printed cHMNs to evaluate the photopolymerization and integration of PBs.

The FTIR spectra of individual components and 3D-printed cHMNs confirmed successful photopolymerization and integration of PBs, illustrated in Figure 3. Characteristic peaks of GAN, Jurymer, PVA, PEGDMA, and LAP were observed, including broad O–H stretching (~3200–3500 cm^−1^), strong C=O stretching (~1700–1730 cm^−1^), and C–O stretching (~1000–1200 cm^−1^), indicating the presence of polymeric backbones and ester linkages [39,40,41]. In the cHMN control (crosslinked sample), the intensity of C=C-related peaks (~1620 cm^−1^) from PEGDMA significantly decreased or disappeared, confirming effective photocrosslinking under UV light [39]. This process was initiated by LAP, a highly efficient photoinitiator that absorbs light (typically at 405 nm) and undergoes cleavage to generate free radicals. These radicals initiate chain polymerization by reacting with the vinyl groups (C=C) of PEGDMA, leading to the formation of a crosslinked polymer network. The persistence of C=O and C–O peaks suggests that the structural backbone remains intact after curing, while slight peak shifts and broadening indicate intermolecular interactions such as hydrogen bonding [42]. These molecular interactions contribute to enhanced mechanical strength, consistent with the observed improvements in hardness and elasticity. Overall, the FTIR results, together with the photocrosslinking mechanism of LAP, confirm the successful fabrication of structurally stable and mechanically robust 3D-printed cHMNs suitable for transdermal drug delivery applications.

### 3.3. Preparation and Characterization of E2V-Loaded Nanoparticles (E2V-NPs)

The physicochemical properties of E2V-NPs demonstrated successful formation via the ionotropic gelation method. The nanoparticles exhibited a mean PS of 698.33 (0.78) nm, indicating formation in the submicron range. Although slightly larger than conventional nanocarriers, this size can be attributed to the polymeric nature of NSCS and the ionic crosslinking with TPP, which typically produces relatively larger and more hydrated particles [28]. The PDI of 0.50 (0.06) suggests a moderately broad size distribution, likely due to the aggregation tendency or heterogeneous nucleation during the gelation process [43]. This increase in particle size can be attributed to the higher loading of E2V, which promotes hydrophobic interactions and swelling within the polymeric matrix, leading to particle expansion. Similar trends were observed in the reference study, where increasing EV concentration resulted in a dramatic increase in particle size, reaching up to 14,041 nm at high drug loading [28]. Optimization of formulation parameters such as polymer concentration or sonication conditions may further improve size uniformity [44]. The ZP of −39.09 (7.32) mV indicated a highly negative surface charge, which contributed to good colloidal stability through electrostatic repulsion, preventing particle aggregation [45]. This negative charge was consistent with the presence of carboxylate groups from NSCS and the ionic interaction with TPP [28]. Importantly, the nanoparticles demonstrated a high entrapment efficiency of 86.87 (0.78)%, indicating effective incorporation of E2V within the polymeric matrix. This high encapsulation may be attributed to hydrophobic interactions between E2V and the naphthyl groups of NSCS, as well as the formation of a dense crosslinked network that limits drug diffusion during preparation.

### 3.4. Morphology Study of 3D-Printed cHMNs Loaded with E2V-NPs

**Figure 4:**
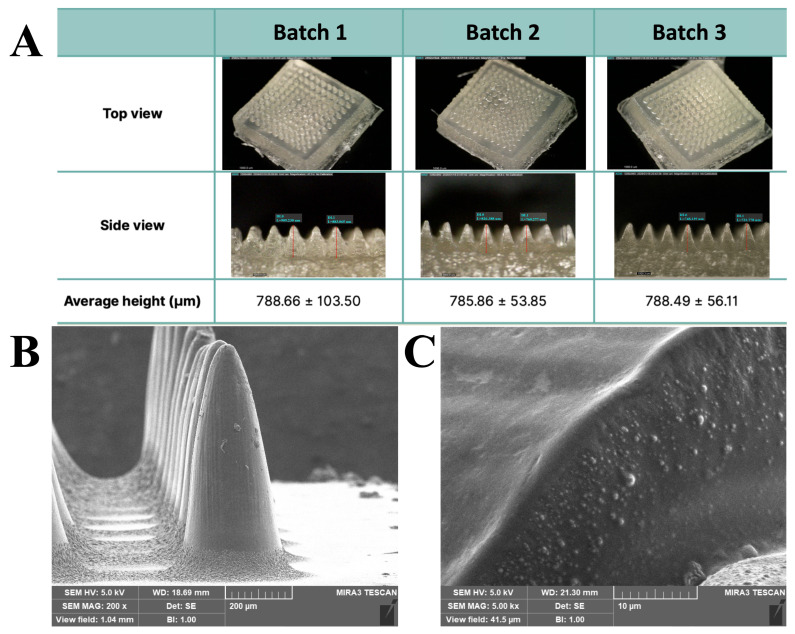
(**A**) Morphological characterization of 3D-printed cHMNs loaded with E2V-NPs: top and side views of microneedle arrays at three independent batches, along with corresponding average needle height measurements (µm). (**B**) SEM images illustrating the surface morphology and tip structure of the 3D-printed cHMNs loaded with E2V-NPs. (**C**) SEM image showing the distribution of E2V-NPs within the cHMN matrix.

The morphology of 3D-printed cHMNs loaded with E2V-NPs was evaluated using digital microscopy from both top and side views, demonstrating well-defined and uniformly distributed microneedle arrays across all replicates, illustrated in Figure 4A. The top-view images revealed a consistent 11 × 11 array with sharp needle tips and minimal structural defects, indicating high printing fidelity of the optimized photopolymerizable bioinks. Side-view analysis confirmed the conical geometry and structural integrity of the microneedles, with clearly distinguishable tips and bases. The measured average heights were 788.66 (103.50) µm, 785.86 (53.85) µm, and 788.49 (56.11) µm for batch 1, 2, and 3, respectively, showing no significant variation between batches and confirming good reproducibility of the printing process. The SEM image in Figure 4B revealed that the 3D-printed cHMNs loaded with E2V-NPs possessed a well-defined conical geometry with smooth surfaces and sharp tips, which was consistent with the uniform morphology observed in the digital microscope images. In addition, Figure 4C showed the presence of nanoscale structures distributed within the cHMN matrix, confirming the successful incorporation of E2V-NPs without disrupting the structural integrity of the microneedles, which was consistent with previous studies [28]. The fabricated cHMNs deviated substantially from the designed height (1500 µm), exhibiting a height shrinkage of approximately 47.4–47.6%, indicating significant dimensional reduction during photocuring and post-processing. This reduction may be attributed to polymer network densification during photocrosslinking, along with water loss and structural contraction during post-processing and drying. In addition, the finite resolution of the LCD 3D printer (22 µm) may have further influenced the fidelity of the printed microneedle geometry, particularly at the needle tip, where small features are more susceptible to rounding or dimensional deviation. However, the cHMNs still maintained a conical shape, and the relatively low standard deviation further supports good dimensional consistency. Overall, the results indicated that incorporation of E2V-NPs did not adversely affect printability or structural precision, and the fabricated cHMNs possessed appropriate geometry and uniformity for effective skin penetration and transdermal drug delivery.

### 3.5. Mechanical Properties of 3D-Printed cHMNs Loaded with E2V-NPs

**Figure 5:**
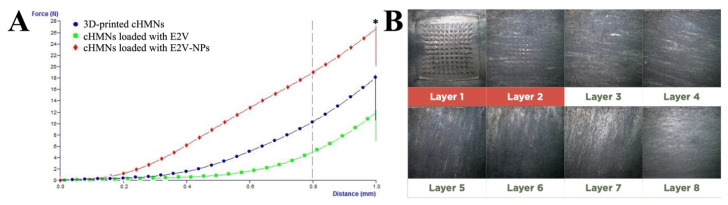
(**A**) Force–displacement curves of 3D-printed cHMNs, cHMNs loaded with E2V, and cHMNs loaded with E2V-NPs obtained using a texture analyzer. (**B**) Penetration study of E2V-NPs-loaded cHMNs using an 8-layer Parafilm^®^ M model (each layer thickness ≈ 130 µm). * indicates significantly higher than others.

The mechanical properties of the 3D-printed cHMNs (Figure 5A) revealed that the unloaded microneedles exhibited a hardness of 18.06 (6.23) N and elasticity of 2.84 (0.20) MPa, indicating adequate baseline mechanical strength. Upon loading with E2V, a reduction in mechanical performance was observed, with hardness decreasing to 11.89 (4.63) N and elasticity to 0.83 (0.12) MPa. This decrease may be attributed to the incorporation of free drug, which could interfere with the polymer network formation and reduce crosslinking efficiency, leading to a less rigid structure [38]. In contrast, the cHMNs loaded with E2V-NPs exhibited the highest mechanical performance, with a hardness of 26.45 (5.23) N and elasticity of 2.97 (0.49) MPa, indicating sufficient rigidity for effective skin insertion while maintaining structural flexibility [46]. Compared to the unloaded cHMNs, the E2V-NPs-loaded system demonstrated enhanced or comparable mechanical properties. This improvement may be attributed to the increased crosslink density and the reinforcing effect of nanoparticles dispersed within the polymer matrix, which can act as physical fillers and enhance stress distribution [37,47,48]. Importantly, the incorporation of E2V-NPs did not compromise the structural integrity of the crosslinked network, suggesting that the developed system maintained mechanical robustness suitable for transdermal microneedle applications.

The penetration study using an 8-layer Parafilm^®^ M model (each layer thickness ≈ 130 µm) further confirmed the insertion capability, represented in Figure 5B. The E2V-NPs-loaded cHMNs achieved 100% penetration in the first layer (~130 µm) and 25.33 (0.58)% in the second layer (~260 µm total depth), with no penetration observed beyond this level (>390 µm). Importantly, this controlled penetration depth suggests that the microneedles can effectively breach the stratum corneum and reach the viable epidermis without penetrating excessively deep into the dermis, thereby minimizing pain and tissue damage [49].

### 3.6. Histological Study

**Figure 6:**
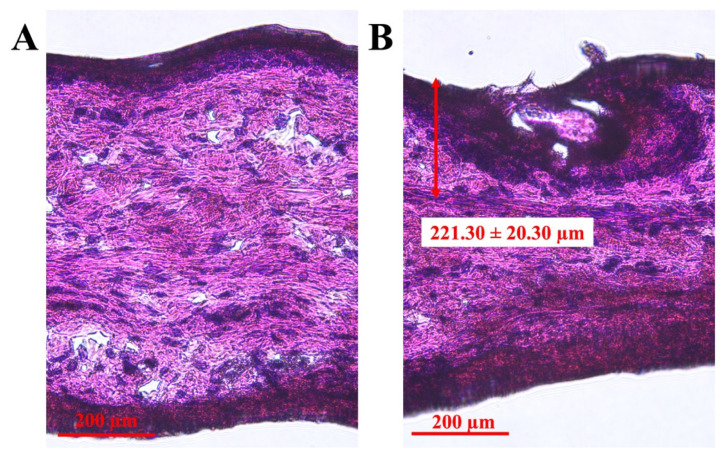
A histological study (40× magnification) of (**A**) control porcine skin and (**B**) porcine skin following application of E2V-NPs-loaded cHMNs, performed to determine the insertion depth.

Figure 6 presents the histological evaluation, demonstrating that the 3D-printed cHMNs loaded with E2V-NPs successfully created microchannels in porcine skin with an average penetration depth of 221.30 (20.30) µm. Notably, the insertion depth observed in porcine skin was slightly lower than that obtained using artificial membrane models. This discrepancy was likely attributed to the intrinsic elasticity and viscoelastic properties of biological skin, which could dissipate the applied force and consequently reduce penetration efficiency [50,51]. Despite this variation, the mechanical strength of the E2V-NPs-loaded cHMNs was sufficient to penetrate the stratum corneum and form well-defined microchannels. This capability was critical for enhancing transdermal drug delivery, as it enabled the drug to bypass the primary skin barrier and facilitated improved permeation into deeper skin layers and systemic circulation [51].

### 3.7. Drug Content Determination

The drug content of E2V in the 3D-printed cHMNs loaded with E2V-NPs was quantified to ensure consistency and accuracy during fabrication. The measured amount of E2V in the cHMN patches was 72.68 (2.23) µg per patch (1 × 1 cm^2^), indicating good content uniformity. These results demonstrated that the E2V loading in the cHMNs was at a satisfactory level. Notably, when compared with commercial transdermal estradiol systems, such as Evra^®^ (Janssen Pharmaceuticals, Inc., Titusville, NJ, USA), which typically deliver approximately 30 µg/cm^2^, the developed cHMNs exhibited a higher drug loading capacity [52]. This suggests that the 3D-printed cHMNs loaded with E2V-NPs had strong potential as an alternative transdermal delivery system with enhanced drug payload.

### 3.8. Degradation Study

**Figure 7:**
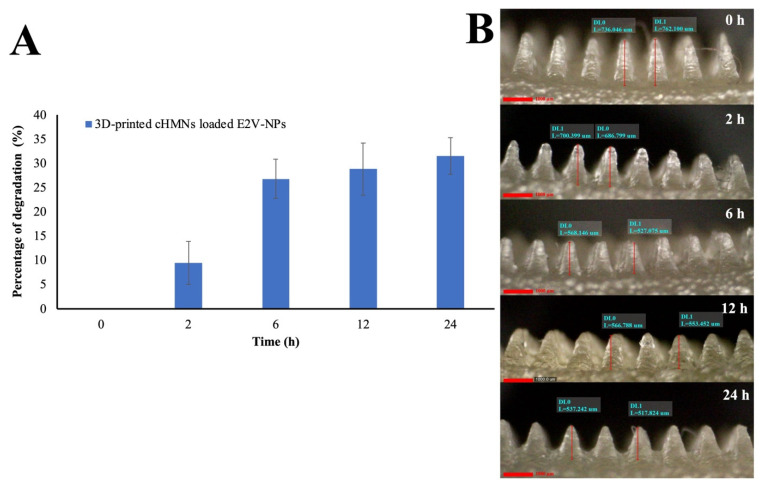
The degradation study of the 3D-printed cHMNs loaded with E2V-NPs: (**A**) the percentage of height reduction and (**B**) the height of the 3D-printed cHMNs loaded with E2V-NPs (47.5× magnification) after being applied on porcine skin at 0, 2, 6, 12, and 24 h.

The degradation behavior of the 3D-printed cHMNs loaded with E2V-NPs was investigated to determine the suitable timeframe for drug release following skin insertion. As shown in Figure 7A, the cHMNs did not undergo complete degradation after 24 h (31.46 (3.78)% of height degradation); however, a gradual increase in the percentage of height degradation was observed over time. Consistently, Figure 7B demonstrated a progressive decrease in cHMN height and swelling throughout the 24 h period. This behavior was attributed to the photocrosslinked polymeric network used in fabricating the cHMNs, which provided a highly stable and densely crosslinked structure. Such a network exhibited resistance to rapid dissolution in aqueous environments, thereby maintaining structural integrity during the observation period. In addition, the degradation profile was influenced by the physicochemical properties of the polymer matrix, including crosslinking density and polymer composition [53,54]. Although the presence of esterase in the simulated interstitial fluid slightly facilitated polymer degradation, its effect was limited [55,56]. Instead, upon contact with the aqueous environment, the cHMNs primarily underwent swelling due to water uptake. This swelling behavior enabled gradual drug release from the polymer matrix while preserving the overall needle structure [49].

### 3.9. In Vitro Drug Release Kinetic Study

**Figure 8:**
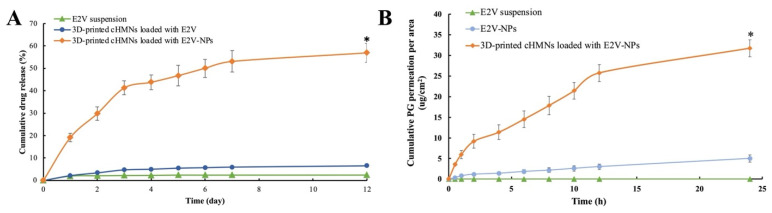
(**A**) In vitro drug release of E2V suspension, 3D-printed cHMNs loaded with E2V, and 3D-printed cHMNs loaded with E2V-NPs. (**B**) In vitro skin permeation of E2V suspension, 3D-printed cHMNs loaded with E2V, and 3D-printed cHMNs loaded with E2V-NPs through porcine skin. * indicates significantly higher than others (*p* < 0.05).

The in vitro drug release profiles of E2V suspension, 3D-printed cHMNs loaded with E2V, and 3D-printed cHMNs loaded with E2V-NPs are presented in Figure 8A. The results demonstrated that the E2V-NPs-loaded cHMNs provided a sustained and controlled drug release over 12 days, reaching a cumulative release of approximately 56.92 (4.27)%, which was significantly higher than the other formulations (*p* < 0.05). The release profile exhibited a gradual increase without an initial burst effect, indicating effective encapsulation of E2V within the nanoparticle–polymer matrix. In contrast, cHMNs loaded with E2V alone showed a markedly lower release (6.54 (0.04)%), suggesting limited diffusion of the poorly soluble drug from the polymer network. Similarly, the E2V suspension exhibited minimal release (2.48 (0.28)%), which was attributed to its low aqueous solubility and limited dissolution in the release medium. The sustained release behavior observed in the E2V-NPs-loaded cHMNs could be explained by a combined swelling- and diffusion-controlled mechanism. Although the cHMNs exhibited limited structural degradation over time, the polymer matrix was capable of absorbing water and swelling, facilitating gradual diffusion of E2V-NPs and dissolved drug into the surrounding medium. Furthermore, the nanoparticle system enhanced drug dispersion and solubilization, thereby improving release efficiency compared to free drug loading [57].

The mathematical models describing the release kinetics of E2V for each formulation are summarized in Table 4. Among the tested models, the Korsmeyer–Peppas model provided the best fit for all formulations, with the highest coefficient of determination (R^2^ = 0.981–0.994), indicating that this model was most suitable for describing the release behavior. The release exponent (n) values were found to be 0.18, 0.35, and 0.78 for E2V suspension, cHMNs loaded with E2V, and cHMNs loaded with E2V-NPs, respectively. The low n values (<0.5) observed for E2V suspension and E2V-loaded cHMNs suggested that drug release was predominantly governed by Fickian diffusion. This behavior was attributed to the limited solubility of E2V and restricted diffusion through the polymer matrix. In contrast, the E2V-NPs-loaded cHMNs exhibited an n value of 0.78, indicating an anomalous (non-Fickian) transport mechanism, where both diffusion and polymer relaxation/swelling (erosion) contributed to drug release [58,59]. This finding was consistent with the swelling behavior observed in the degradation study, where the polymer matrix absorbed water and facilitated sustained drug diffusion without complete structural breakdown. Overall, the incorporation of E2V-NPs into the cHMN system altered the release mechanism from purely diffusion-controlled to a combined swelling–diffusion process, resulting in improved and sustained drug release performance.

### 3.10. In Vitro Skin Permeation and Drug Accumulation Study

The in vitro skin permeation profiles of E2V suspension, E2V-NPs, and 3D-printed cHMNs loaded with E2V-NPs are presented in Figure 8B. The E2V suspension showed negligible permeation, with both flux and cumulative permeation at 24 h (Q_24_/area), indicating poor transdermal delivery due to its low aqueous solubility and the barrier function of the stratum corneum. In contrast, E2V-NPs exhibited enhanced permeation, with a flux of 0.3417 (0.0727) µg/cm^2^/h and a Q_24_/area of 5.01 (0.86) µg/cm^2^, demonstrating that nanoparticle formulation improved drug solubility and diffusion across the skin [6,60]. However, the overall permeation remained limited, likely due to the intact skin barrier. Notably, the 3D-printed cHMNs loaded with E2V-NPs showed significantly higher permeation, with a flux of 10.8135 (4.5505) µg/cm^2^/h and a Q_24_/area of 12.94 (2.06) µg/cm^2^. This substantial increase confirmed the effectiveness of the cHMN system in enhancing transdermal delivery. The improved permeation was attributed to the formation of microchannels by the microneedles, which bypassed the stratum corneum and facilitated direct drug transport into deeper skin layers [61].

In addition to permeation, the amount of E2V accumulated in the porcine skin after 24 h was quantified. The E2V suspension showed the lowest skin accumulation (13.11 (0.05) µg), which was consistent with its negligible permeation, indicating limited ability to penetrate and retain within the skin layers. In contrast, E2V-NPs significantly increased skin accumulation (27.02 (0.39) µg), suggesting that nanoparticle encapsulation improved drug solubility and promoted interaction with the skin microenvironment, thereby enhancing drug retention in both the epidermis and dermis [6,60]. Notably, the 3D-printed cHMNs loaded with E2V-NPs exhibited the highest skin accumulation (38.55 (0.10) µg), in agreement with the enhanced permeation results. This improvement was attributed to the formation of microchannels by the cHMNs, which bypassed the stratum corneum and enabled direct delivery of E2V-NPs into deeper skin layers. Furthermore, the combination of MNs-assisted delivery and nanoparticle encapsulation provided a synergistic effect, involving both physical disruption of the skin barrier and sustained drug release from the swollen polymer matrix. This dual mechanism not only enhanced transdermal flux but also increased drug retention within the skin [28,62].

### 3.11. In Vitro Cytotoxicity Study

**Figure 9:**
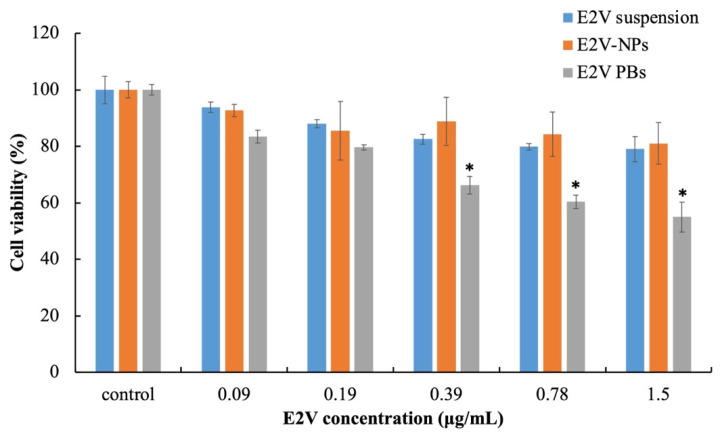
Effects of E2V in each formulation on NHF cell viability. Each bar represents the mean (SD) (*n* = 5). * indicates statistical significance compared to the others (*p* < 0.05).

The cytocompatibility of E2V suspension, E2V-NPs, and E2V-loaded PBs toward NHFs is presented in Figure 9. All formulations maintained high cell viability (~100%) in the control group. At the lowest concentration (0.09 µg/mL), cell viability remained above 90% for E2V suspension and E2V-NPs, while a slight reduction was observed for E2V-loaded PBs (~83%). As the concentration increased, a gradual decrease in cell viability was observed for all formulations. E2V suspension consistently exhibited the highest viability across all concentrations, remaining above ~79% even at 1.5 µg/mL. E2V-NPs showed a moderate reduction in viability at higher concentrations but still maintained acceptable cell viability (~80% at 1.5 µg/mL). These results indicated that both E2V and E2V-NPs exhibited favorable cytocompatibility profiles.

In contrast, E2V-loaded PBs demonstrated a more pronounced decrease in cell viability, with values dropping to ~60% at 0.78 µg/mL and ~55% at 1.5 µg/mL, indicating a concentration-dependent cytotoxic effect. This increased cytotoxicity was likely associated with the presence of the photoinitiator (LAP), which has been reported to exhibit dose-dependent cytotoxicity due to the generation of reactive species during photopolymerization. Previous studies have demonstrated that higher concentrations of LAP can reduce cell viability through oxidative stress mechanisms [63]. However, it was important to note that the LAP concentration used in this study (0.5% *w*/*w*) was considered safe for photocrosslinking applications, particularly in solidified systems. In the 3D-printed cHMNs, LAP was immobilized within the crosslinked polymer network, which limited its direct interaction with cells and reduced its cytotoxic potential. This was consistent with previous reports indicating that optimized LAP concentrations could achieve effective crosslinking while maintaining acceptable biocompatibility [64,65].

## 4. Conclusions

This study successfully developed and optimized PBs for the fabrication of cHMNs using LCD-based 3D printing for enhanced transdermal delivery of E2V-NPs. Notably, this work represents one of the first systematic applications of a DoE approach to optimize PB formulations specifically for 3D-printed cHMNs, enabling a rational and data-driven balance between printability, mechanical integrity, and microneedle performance. This approach represents an important step toward more systematic and reproducible fabrication of 3D-printed microneedle systems. The optimized E2V-NPs-loaded cHMNs successfully addressed key limitations associated with conventional transdermal delivery of poorly soluble drugs, including insufficient permeation, limited skin deposition, and the need for sustained drug release. By combining nanoscale drug carriers with a structurally robust microneedle platform, the developed system enhanced drug transport across the skin while maintaining suitable mechanical performance for skin insertion. These findings support the feasibility of using 3D-printed cHMNs as an advanced transdermal delivery strategy for hormone therapy and other poorly soluble therapeutic agents.

Overall, the present work highlights the broader significance of combining pharmaceutical formulation science with digital manufacturing and statistical optimization. The proposed platform offers a promising pathway for the future development of personalized, scalable, and next-generation transdermal drug delivery systems. Further studies focusing on long-term safety, in vivo pharmacokinetic performance, and clinical translation will be important to fully establish the therapeutic potential of this technology.

## Figures and Tables

**Table 1:** The Box–Behnken design (BBD) results of photopolymerizable bioinks (PBs).

Factor 1	Factor 2	Factor 3	Response 1	Response 2	Response 3	Response 4
X_1_: GAN (%*w*/*w*)	X_2_: Jurymer (%*w*/*w*)	X_3_: PVA (%*w*/*w*)	Y_1_: Viscosity (cP)	Y_2_: Exposure Time (sec)	Y_3_: Hardness (N)	Y_4_: Elasticity (MPa)
1	1	3	313	104	0.8144	0.3151
1	3	1	150.5	100	0.2917	0.072
1	3	5	11,047	194	3.259	1.841
1	5	3	1347	116	1.676	1.089
5.5	1	1	25.21	85	1.149	0.1551
5.5	1	5	25,928	258	5.1259	2.7182
5.5	3	3	2025	119	2.1771	1.792
5.5	3	3	4088	129	2.897	2.2132
5.5	3	3	3548	123	3.022	2.342
5.5	5	1	139.23	98	1.456	0.5332
5.5	5	5	10,345	189	8.342	3.5412
10	1	3	14,395	204	18.8321	3.3413
10	3	1	6842	135	11.1081	2.3413
10	3	5	39,673	300	23.426	5.4201
10	5	3	14,050	201	19.342	3.949

**Table 2:** Summary of the ANOVA and multiple regression analysis for BBD.

Responses ^#^		Fit Summary			
	Model	*p*-Value	LACK OF FIT	R^2^	Recommend
Viscosity	Reduced Quadratic	<0.05	0.1051	0.9702	Suggested
Exposure time	Reduced Quadratic	<0.05	0.1298	0.9837	Suggested
Hardness	Reduced Quadratic	<0.05	0.1053	0.9817	Suggested
Elasticity	Reduced Quadratic	<0.05	0.9009	0.9907	Suggested
Multiple regression equation model (coded equation)					
Y_1_ = 2908.69 + 7762.81X_1_ − 1847.50X_2_ + 9979.51 X_3_ + 5483.63X_1_X_3_ − 3924.26X_2_X_3_ + 4851.30X_1_^2^ + 6434.41X_3_^2^					
Y_2_ = 126.08 + 40.75X_1_ − 5.87X_2_ + 65.38X_3_ + 17.75X_1_X_3_ − 20.50X_2_X_3_ + 28.37X_1_^2^ + 29.62X_3_^2^ Y_3_ = 3.45 + 8.33X_1_ + 3.27X_3_ + 2.34X_1_X_3_ + 6.39X_1_^2^					
Y_4_ = 2.07 + 1.47X_1_ + 0.32X_2_ + 1.30X_3_ + 0.33X_1_X_3_ + 0.35X_1_^2^ − 0.31X_2_^2^					

^#^ X_1_: Gantrez S97 (%*w*/*w*); X_2_: Jurymer (%*w*/*w*); X_3_: PVA (%*w*/*w*); Y_1_: Viscosity (cP); Y_2_: Exposure time (sec); Y_3_: Hardness (N); Y_4_: Elasticity (MPa).

**Table 3:** Optimal formulation of the photopolymerizable bioinks (PBs) predicted by the model and validated experimentally.

Factors	Goal	R_p_	R_e_	Desirability	95% Prediction Interval (PI)
**Input Factors**					
X_1_: Gantrez S97 (%*w*/*w*)	In range	10.00	10.00	0.659	
X_2_: Jurymer (%*w*/*w*)	In range	3.20	3.20		
X_3_: PVA (%*w*/*w*)	In range	2.04	2.04		
**Output Factors**					
Y_1_: Viscosity (cP)	Minimize	9569.12	9427.60 (392.8)		In range PI
Y_2_: Exposure time (s)	Minimize	162.34	176.67 (11.57)		In range PI
Y_3_: Hardness (N)	Maximize	15.48	14.86 (1.23)		In range PI
Y_4_: Elasticity (MPa)	Maximize	3.162	2.84 (0.20)		In range PI

**Table 4:** The E2V profile release kinetic model, including the coefficient of determination (R^2^), constant of regression equations (k) and regression equations (* indicates the highest R^2^).

Model	E2V Suspension				3D-Printed cHMNs Loaded with E2V				3D-Printed cHMNs Loaded with E2V-NPs			
	R^2^	k	n	Regression Equation	R^2^	k	n	Regression Equation	R^2^	k	n	Regression Equation
Zero order	0.352	0.01	-	y = 0.005x + 1.50	0.706	0.02	-	y = 0.02x + 2.17	0.706	0.177	-	y = 0.177x + 18.20
First order	0.354	−2 × 10^−05^	-	y = −2 × 10^−05^x + 1.99	0.888	−0.001	-	y = −0.001x + 1.20	0.795	−0.001	-	y = −0.001x + 1.91
Higuchi	0.668	0.13	-	y = 0.13x + 0.86	0.985	0.503	-	y = 0.50x − 0.03	0.935	0.614	-	y = 3.614x + 4.56
Hixson and Crowell	0.352	0.00	-	y = 8 × 10^−05^x + 0.024	0.885	0.001	-	y = 0.001x + 0.017	0.766	0.004	-	y = 0.004x + 0.32
Korsemeyer and Peppas	0.981 *	-	0.18	y = 0.18x + 0.009	0.994 *	-	0.35	y = 0.35x − 0.004	0.981 *	-	0.78	y = 0.78x + 0.04

## Data Availability

The original contributions presented in this study are included in the article. Further inquiries can be directed to the corresponding author.
